# Life-Long Hyperbilirubinemia Exposure and Bilirubin Priming Prevent *In Vitro* Metabolic Damage

**DOI:** 10.3389/fphar.2021.646953

**Published:** 2021-03-12

**Authors:** Annalisa Bianco, Serena Pinci, Claudio Tiribelli, Cristina Bellarosa

**Affiliations:** ^1^ Italian Liver Foundation (FIF), Trieste, Italy; ^2^ Department of Life Sciences, University of Trieste, Trieste, Italy

**Keywords:** Bilirubin, life-long hyperbilirubinemia, metabolic damage, apoptosis, inflammation, fibrosis, ER-stress

## Abstract

**Background:** Unconjugated bilirubin (UCB) is more than the final product of heme catabolism. Mildly elevated systemic bilirubin concentrations, such as in Gilbert syndrome (GS), protect against various oxidative stress-mediated and metabolic diseases, including cardiovascular disease, type 2 diabetes mellitus, metabolic syndrome, cancer, and age-related disease. The Gunn rat is an animal model of hereditary hyperbilirubinemia widely used in assessing the effect of high serum bilirubin concentration in various organs. The present work aims to understand if life-long hyperbilirubinemia and bilirubin-priming might contribute to protection against atherosclerosis and diabetic nephropathy (DN) at the cellular level.

**Methods:** Primary aortic endothelial cells and podocytes obtained from hyperbilirubinemic homozygous jj and normobilirubinemic heterozygous Nj Gunn rats were exposed to Palmitic Acid (PA) and Angiotensin II (Ang II), respectively, and the effects on cell viability and the activation of damage-related metabolic pathways evaluated. Results were validated on immortalized H5V and HK2 cells exposed to damage after UCB pretreatment.

**Results:** In both primary cell models, cells obtained from jj Gunn rats showed as significantly higher than Nj Gunn rats at any dose of the toxic agent. Reduction in CHOP expression and IL-6 release was observed in jj primary aortic endothelial cells exposed to PA compared to Nj cells. The same occurred on H5V pretreated with Unconjugated bilirubin. Upon Ang II treatment, primary podocytes from jj Gunn rats showed lower DNA fragmentation, cleaved caspase-3, and cleaved PARP induction than primary podocytes from Nj Gunn rats. In HK2 cells, the induction by Ang II of HIF-1α and LOXl2 was significantly reduced by UCB pretreatment.

**Conclusion:** Our data suggest that in models of atherosclerosis and DN life–long hyperbilirubinemia exposure or bilirubin-priming significantly contribute to decrease the injury by enhancing thecellular defensive response,

## Introduction

Unconjugated bilirubin (UCB) is the final product of the heme catabolic pathway in the intravascular compartment. UCB is transported in the blood tightly bound to serum albumin before being taken up by the hepatocytes, where it is conjugated. The UCB fraction unbound to albumin (so-called free bilirubin, Bf) represents less than 0.1% and determines the biological properties of this pigment (Ahlfors et al., 2009). Bilirubin behavior in a human body has two faces, similar to the Janus Bifrons, a Roman god (Bianco et al., 2020). Elevated serum UCB concentration, and in particular the Bf fraction, exposes newborns to the risk of neurotoxicity (Watchko and Tiribelli, 2013). Conversely, mildly elevated systemic bilirubin concentrations, such as in Gilbert syndrome (GS) (Bosma et al., 1995), protect against various oxidative stress-mediated and metabolic disorders including cardiovascular disease, type 2 diabetes mellitus, metabolic syndrome, cancer, and age-related disease (Wagner et al., 2015; Vitek et al., 2018). These broad metabolic effects depend on the potent antioxidant activities, anti-inflammatory and immunomodulatory effects (Gazzin et al., 2016), and the recently described endocrine activity of UCB (Hinds Terry and Stec David, 2018; Hinds and Stec, 2019; Creeden et al., 2020; Vítek, 2020).

The partial deficiency of hepatic bilirubin UDP glucuronosyl transferase (UGT1A1) enzyme described in Gilbert patients, increases the unconjugated bilirubin level from 10µM (0.6mg/dl) to 20–70µM (1–5mg/dl) without any sign of liver damage (Vítek and Ostrow, 2009; Wagner et al., 2015). The Gunn rat is a model of hereditary hyperbilirubinemia widely used in assessing the effect of high serum bilirubin concentration in various organs. UCB concentration in hyperbilirubinemic homozygous jj Gunn rats (jj) serum (approximately from 2.42 to 7.36mg/dl) (Boon et al., 2012; Vianello et al., 2018) overlaps with elevated bilirubin concentrations seen in Gilbert subjects (Vítek et al., 2002; Bulmer et al., 2008; Boon et al., 2012). The UCB concentration in various organs and tissues of adult jj Gunn rats is higher than the normobilirubinemic heterozygous Nj Gunn rats (Nj) littermates. The UCB tissue content differs 131 fold in the myocardium (12.3ng/mg of tissue in jj vs. 0.093ng/mg of tissue in Nj) and 144 fold in the kidney (19.3ng/mg of tissue in jj vs. 0.134ng/mg of tissue in Nj) (Zelenka et al., 2008). The hyperbilirubinemia shown by jj Gunn rat is associated with marked anti-inflammatory (Wang et al., 2004), antiproliferative (Ollinger et al., 2005), antihypertensive (Pflueger et al., 2005), blood lipid-modulating properties (Wallner et al., 2013) and with fewer signs of cellular senescence (Zelenka et al., 2016). Moderate hyperbilirubinemia was demonstrated to lower (Ang II)–dependent hypertension by a mechanism that is partially dependent on the inhibition of superoxide production *in vivo* (Stec et al., 2013).

Atherosclerosis represents the major cause of cardiovascular diseases and it is caused by a combination of immune and inflammatory conditions leading to arterial wall injury (Zhang and Kaufman, 2008; Kang et al., 2014). The accumulation of cholesterol and free fatty acids (FFAs) in the ER membranes of macrophages causes calcium release, UPR activation, and CHOP-induced apoptosis (Feng et al., 2003). Palmitic acid (PA) plays an important role in the development of atherosclerosis (Wu et al., 2014) due to its abundance as saturated fatty acid in FFAs (Lambertucci et al., 2008). The accumulation of PA in macrophages activates different transcriptional factors such as NF-κB, thereby inducing the expression of genes encoding inflammatory cytokines including TNF-α and IL-6 (Zhang and Kaufman, 2008; Mazzone et al., 2009).

Diabetic nephropathy (DN) is a complication of diabetes mellitus and it is the leading cause of end-stage renal disease. The final effects of DN involve endothelial injury, tubulointerstitial fibrosis, and podocyte detachment and apoptosis (Maezawa et al., 2015). Angiotensin II (Ang II) has been commonly used in *in vitro* modeling of DN as a pathophysiological mediator that mimics the disease (Slyne et al., 2015). HIF-1α was recently identified as a promoter of kidney fibrosis (Haase, 2012) and has been demonstrated that its activation stimulates collagen accumulation and inflammatory cell recruitment in experimental model of CKD (Higgins et al., 2007; Kimura et al., 2008; Wang et al., 2011). While hypoxia is the main stimulus for HIF activation, Ang II have also been shown to activate HIF-1α (Patten et al., 2010). LOXl2 is considered an element of pro-fibrotic HIF-1α pathway contributing to tubulointerstitial fibrosis development (Schietke et al., 2010). The early pathological changes of DN include podocyte apoptosis (Kume and Koya, 2015). Cleavage of PARP by caspase-3 is considered a hallmark of apoptosis (Jagtap and Szabó, 2005).

The present work aimed to understand if life-long hyperbilirubinemia and bilirubin-priming might have a protective effect in *in vitro models* of atherosclerosis and DN. The use of cellular models might help us to unravel the mechanisms associated with protection from renal injury and metabolic syndrome. Cells obtained from hyperbilirubinemic jj and normobilirubinemic Nj Gunn rats were exposed to damage in the absence of UCB. ER-stress and inflammation activation were evaluated on primary aortic endothelial cells exposed to PA to mimic atherosclerosis. Cell death by apoptosis was studied on primary podocytes exposed to Ang II to mimic diabetic nephropathy. Results were also validated on immortalized cell lines pretreated with UCB (bilirubin-priming) and then exposed to damage.

## Materials and Methods

### Primary Cultures of Rat Podocytes and Aortic Endothelial Cells

Rats used in the project were born in the animal facility of the University of Trieste and animal handling was approved by the Ethics Committee for Animal Experimentation (OPBA) of the University of Trieste (NO1487BEL19) in compliance with the Italian regulation (D.L.vo 26/2014) and the Directive 2010/63/EU of the European Parliament. Hyperbilirubinemic jj and normobilirubinemic Nj Gunn rats were obtained by breeding a male homozygote (jj) with a female heterozygote (Nj). Rats were anesthetized by intraperitoneal injection of Zoletin (60mg/Kg) and Xylazine (40mg/Kg) and successively sacrificed through decapitation. 90% of rats used were male, but we did not distinguish between males and females, Rats were sacrificed when reached a weight around 200gr, corresponding to 8weeks for males and 10weeks for females..

The kidneys were extracted and conserved in d-glucose (5.5mM) Dulbecco’s Modified Eagle’s Medium (DMEM) and Ham’s F12 medium (Euroclone S. p.A., Milano, Italy) in the ratio 1:1 on ice; Bowman’s capsule was removed by pliers (Rush et al., 2018) and the cortical region of the organ was cut into thin strips. Glomeruli were collected in 50ml DMEM/F12 solution using filters with different sizes (80, 100, and 300 meshes) (Sigma-Aldrich, St. Louis MO, United States) and a pestle. They were then centrifuged at 136g for 5minat room temperature. The pellet was seeded in the 25cm^2^ culture flasks pre-coated with collagen type IV (Sigma-Aldrich, St. Louis MO, United States). Glomeruli were left in the incubator at 37°C in 5% CO_2_ humidified atmosphere in a medium composed by DMEM low glucose and Ham’s F12 media (1:1) supplemented with a final concentration of decomplemented 10% (v/v) FBS, 1% penicillin/streptomycin solution (penicillin G (100U/mL), streptomycin (100mg/ml)), l-glutamine (2mmol/L), bovine insulin (5mg/ml), 5μg/ml holo-transferrin (5mg/ml), sodium selenite (5ng/ml) and hydrocortisone (5ng/ml) (Sigma-Aldrich, St. Louis MO, United States) (Zennaro et al., 2014). After 4/5days of incubation podocytes began to emerge and grow out from the glomeruli and, once reached 70–80% of confluence, they were detached using trypsin-EDTA (Euroclone S.p.A., Milano, Italy) and scraper, and collected using a 40-mesh filter. Finally, they were seeded in the 25cm^2^ culture flasks pre-coated with collagen type IV (Sigma-Aldrich, St. Louis MO, United States).

The aorta was extracted and conserved in Human Endothelial serum-free medium GIBCO (Life Technologies, Monza, Italy). The aorta (approximately 2cm long) was cleaned from fat tissue, cut into two halves longitudinally, and digested by collagenase type II (2mg/ml) (Worthington Industries, OH, United States). After collagenase treatment, endothelial cells removed from the aorta were collected by centrifugation and suspended in 10ml of Human Endothelial medium GIBCO (Life Technologies, Monza, Italy) supplemented with a final concentration of 10% FBS, EGF (10ng/ml), bFGF 20ng/ml, and 1% penicillin/streptomycin solution (penicillin G (100U/mL) in 5 wells (2ml/well) of 6-multiwell coated with gelatin. After 1h of incubation at 37°C, the supernatant containing endothelial cells was re-seeded in another well. Then aortic endothelial cells were isolated and characterized by magnetic beads coated with CD31 (BD Biosciences, San Jose, CA), a characteristic marker for endothelial cells (Liu et al., 2008).

### Immortalized Cell Culture

HK2, an immortalized human proximal tubular epithelial cell line (kindly provided by Prof. R. Bulla, Department of Life Sciences, University of Trieste), was cultured in DMEM low glucose and Ham’s F12 media (1:1) supplemented with a final concentration of decomplemented 5% (v/v) FBS, 1% penicillin/streptomycin solution (penicillin G (100U/mL), streptomycin (100mg/ml)), l-glutamine (2mmol/L), bovine insulin (5mg/ml), holo-transferrin (5mg/ml), sodium selenite (5ng/ml), hydrocortisone (5ng/ml), EGF (10ng/ml), T3 (5pg/ml), and PGE (15pg/ml).

H5V, murine heart endothelial cells transformed by polyomavirus middle T-antigen (kindly provided by Istituto Mario Negri, Milan, Italy) were grown in DMEM low glucose containing a final concentration of 10% (v/v) FBS, 1% penicillin/streptomycin solution (penicillin G (100U/mL), streptomycin (100mg/ml)), and l-glutamine (2mmol/L).

Once reached 80% of confluence, both the cell lines were employed for the experimental studies as described below.

### Treatments

#### UCB Treatment

UCB (Sigma-Aldrich, St. Louis MO, United States) was purified as previously described (McDonagh and Assisi, 1972) and dissolved in DMSO to reach a final concentration of 6mM. To reach the final concentrations desired, UCB was serially diluted with DMSO and then with a growth medium. All UCB treatments were performed in the presence of BSA 30µM. The solvent was always 0.1% (v/v) in all bilirubin concentrations used. DMSO 0.1% was used to treat control cells. H5V and HK2 UCB pretreatment were performed in cell culture medium in the presence of BSA 30μM with total UCB concentrations of 1.25 and 2.5μM. According to Roca et al. (2006), these concentration correspond to a Bf < 15nM and = 15nM, respectively. These concentrations of Bf were chosen since they are similar to the plasma Bf levels found in humans with mild unconjugated hyperbilirubinemia (Jacobsen and Wennberg, 1974; Nelson et al., 1974).

#### Angiotensin II Treatment

Human Angiotensin II (Sigma-Aldrich, St. Louis MO, United States) was reconstituted with phosphate-buffered saline (PBS) at the concentration of 1mg/ml. The range of angiotensin II concentration used in the experiments ranged from 0.01µM up to 10µM and all treatments required 24h (Chen et al., 2017).

#### Palmitic Acid Preparation and Treatment

Palmitic acid (PA) 0.1M stock solutions were prepared by dissolving fatty acid-free (FFAs) in DMSO. The cells were exposed for 24h. Since albumin played a crucial role in determining the FFAs available concentration, the BSA concentration was kept fixed at 75µM. The FFAs were complexed with bovine serum albumin at a different molar ratio. The medium containing BSA 75µM was aliquoted to set up various BSA/PA ratios (1:1, 1:2, 1:3, 1:4, 1:5, 1:6). DMSO solution was used as control and its final concentration never exceeded 0.1%.

### Cytotoxicity Test (PI) and Metabolic Activity Test (MTT)

Both the cytotoxicity test and metabolic activity test were performed in black 96-well plates. Propidium iodide (PI) (Sigma-Aldrich, St. Louis MO, United States) was solubilized in PBS to a final concentration of 50μg/ml. After 60min of incubation at 37°C, the initial fluorescence intensity due to the dead cells was measured using a multiplate reader (EnSpire 2300, PerkinElmer, MA, United States). The excitation and the emission wavelengths were 530 and 620nm, respectively. After the measurement, Triton X-100at a final concentration of 0.6% was added to each well to permeabilize the cells and let PI label cell nuclei. After 30min of incubation on ice, fluorescence intensity was re-measured to obtain a value corresponding to the total cells. The percentage of dead cells was calculated as the proportion of fluorescence intensity of dead cells to that of total cells.

Metabolic activity was determined by assessing the reduction of 3 (4,5-dimethyl thiazolyl-2)-2,5 diphenyl tetrazolium (MTT, Sigma-Aldrich, St. Louis MO, United States) to formazan by succinate dehydrogenase, a mitochondrial enzyme. In brief, a stock solution of MTT was prepared in PBS (5mg/ml) and then diluted to 0.5mg/ml in the cell medium. Cells were incubated with the cell medium containing MTT for 1hat 37°C. At the end of the incubation time, the insoluble formazan crystals were dissolved in 100µL of DMSO. Absorbance was determined at 562nm by using a multiplate reader (EnSpire 2300, PerkinElmer). The results were expressed as the percentage of control cells not exposed to UCB, which were considered as being 100% viable. It is important to note that MTT test is a quantitative colorimetric assay to detect the survival and proliferation rate of living cells (Mosmann, 1983).

### Total Protein Extractions, Quantification, and Western Blot Analysis

Cells were treated when they reached a confluence of approximately 80%. Total proteins were extracted by lysing the cells in an ice-cold cell lysis buffer (Cell Signaling Technology, MA, United States). The protein concentration was determined by the bicinchoninic acid protein assay (BCA) according to manufacturer’s instructions. Equal amounts of protein were separated by SDS-polyacrylamide gel electrophoresis (SDS-PAGE) and transferred to PVDF membranes (Bio-Rad Laboratories, CA, United States). Membranes were blocked in 4% milk or 4% BSA in T-TBS (0.2% Tween 20, 20 mMTris–HCl (pH 7.5) and 500 mMNaCl) and incubated overnight at 4°C with the primary antibodies: Anti-HIF-1α antibody (GeneTex, CA, United States), Anti-LOXl2 antibody (CUSABIO Technology LLC, TX, United States), Anti-PARP antibody (Cell Signaling Technology, MA, United States), Anti-CHOP antibody (Invitrogen, Carlsbad, United States) Anti-Caspase-3antibody (Pro and Cleaved forms) (Thermo Fisher Scientific, MA, United States) and antiα-Actin (Sigma-Aldrich, St. Louis MO, United States). Membranes were then incubated with anti-rabbit or anti-mouse secondary antibodies (Dako Laboratories, CA, United States). Protein bands were detected by peroxide reaction using ECL Plus Western Blot detection system solutions (ECL Plus Western Blot detection reagents, GE-Healthcare Bio-Sciences). The relative intensity of protein bands was scanned and analyzed using the C-DiGit® Blot Scanner—LI-COR (Biosciences). The optical density of the bands of interest was normalized to α-Actin and represented as relative to DMSO-treated cells.

### Immunofluorescence

Podocytes were seeded onto appropriate glass coverslips pre-coated with poly-l-lysine (Sigma Aldrich, United States) and fixed with 4% paraformaldehyde. A permeabilization step was performed using 0.5% Triton X-100 for 5min. After being blocked with 5% bovine serum albumin (BSA) in PBS-T + 0.1% Tween 20 for 60minat room temperature, the coverslips were incubated with the primary antibody rabbit anti-nephrin (Sigma-Aldrich, St. Louis MO, United States) or anti-Cleaved Caspase-3 antibody (Elabscience, TX, United States) overnight at 4°C. The coverslips were washed thoroughly in PBS and incubated with Alexa Fluor 546 goat anti-rabbit (Invitrogen, Carlsbad, CA, United States) in the dark for 1hat room temperature. After appropriate washes, cell nuclei were stained with Hoechst 33,328 (Sigma-Aldrich, St. Louis MO, United States) at room temperature. Cells were mounted with Fluorescent Mounting Media (Calbiochem, San Diego, CA, United States) and images were acquired using a Leica DM 2000 microscope equipped with an appropriate filter to cover both the excitation and emission wavelengths of Alexa Fluor 546 and Hoechst 33,328. In each experimental setting, images were captured with identical light exposure parameters and aperture settings.

### TUNEL Assay

For *in situ* detection of DNA fragmentation, the Click-iT ™ Plus TUNEL Assay was used following the manufacturer’s protocol (Thermo Fisher Scientific, MA, United States).

### IL-6 ELISA

The supernatants of H5V and Aortic endothelial primary cells were collected and subjected to ELISA analysis using Mouse IL-6 ELISA KIT (Diaclone, Besançon, Francia) and Rat IL-6 ELISA KIT (Thermo Fisher Scientific, MA, United States) respectively, following the manufacturer’s instructions.

### Statistical Analysis

GraphPad Prism 5 software (GraphPad, San Diego, CA, United States) was used to perform statistical analysis. A statistically significant difference between two data sets was assessed by unpaired two-tailed Student’s t-test. Data were obtained from at least three independent experiments and were expressed as mean ± SD. Significance was graphically indicated as follows: *p < 0.05, **p < 0.01, ***p < 0.001.

## Results

### PA and Angiotensin II Impact on Cell Viability in Nj and jj Primary Cells

**FIGURE 1:**
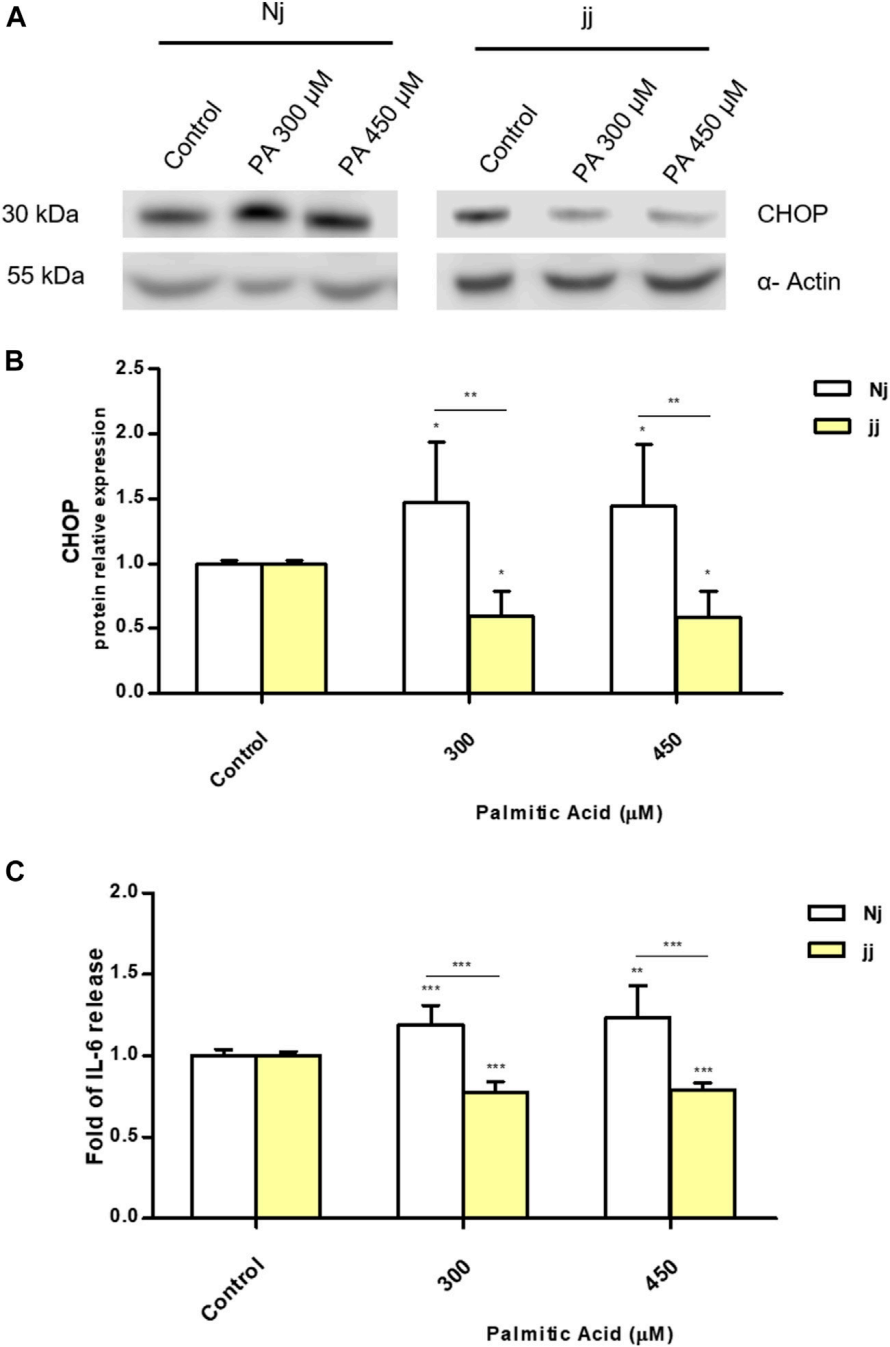
Effect of PA and Ang II treatments on cell viability (MTT test) in normobilirubinemic (Nj) and hyperbiliruninemic (jj) primary cells. **(A)** Aortic endothelial primary cells were exposed to increasing PA concentrations (from 75 to 450µM in the presence of 75µM BSA) for 24h. Cells treated with complete growth medium were considered 100% of viability. Data were expressed as mean ± SD of six independent experiments. **(B)** Primary podocytes were exposed to the Ang II 0.01, 0.1, and 1µM for 24h. Cells treated with complete growth medium were considered 100% of viability. Data were expressed as mean ± SD of three independent experiments. *p < 0.05, **p < 0.01, ***p < 0.001.

Aortic endothelial cells harvested from Nj and jj Gunn rats were treated for 24h with increasing doses of PA ranging from 75μM up to 450μM in the presence of 75μM BSA. As shown in Figure 1A, upon 75μMPA Nj cells showed a 30% reduction of their viability of as compared to 15–20% jj cells ’. A significantly higher (*p < 0.05, ***p < 0.001) viability of jj-derived was found at any PA concentration.

Primary podocyte characterization by immunofluorescence indicated a highly elevated level of nephrin protein with a plasma membrane and perinuclear co-localization (Supplementary Figure S1). The viability of primary podocytes exposed to Ang II 0.01, 0.1, and 1μM for 24h (Figure 1B) was significantly (**p < 0.01, ***p < 0.001) reduced at any Ang II concentration (20–25% vs. 10–15% in Nj and jj podocytes.)

**FIGURE 2:**
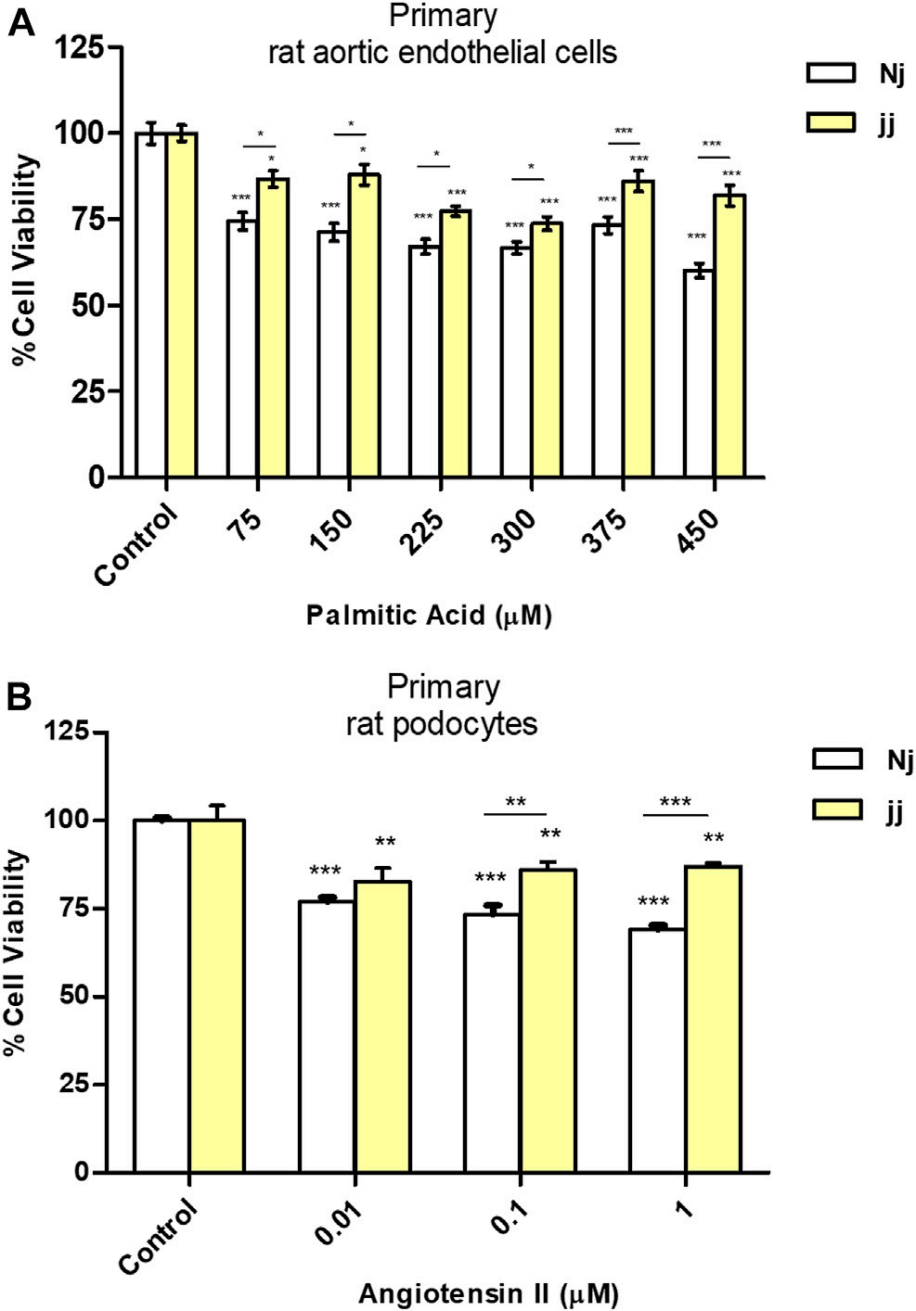
The effect of UCB pretreatment in PA-induced damage on immortalized H5Vcells. H5Vcells were pre-incubated with UCB 1.25 or 2.5μM or DMSO 0.1% (as control) in the presence of BSA 30µM for 16h. At the end of incubation, UCB was removed and H5Vcells were treated with PA 300μM for 24h. **(A)** UCB effect on cell viability was measured by MTT. The viability of control cells was considered 100%. **(B)** UCB effect on cell death detected by PI assay. The percentage of dead cells was calculated as the proportion of fluorescence intensity of dead cells to that of total cells. **(C)** Representative Western Blot for CHOP and α-Actin. **(D)** The optical density of each band was normalized to α-Actin and represented as relative to control cells. **(E)** ELISA quantification of IL-6 release in the cell medium. Results were expressed as relative to control cells release, considered as one. Data were expressed as mean ± SD of at least three independent experiments. *p < 0.05, **p < 0.01, ***p < 0.001.

The effect of bilirubin priming on cell viability was also evaluated on immortalized cell lines. Heart endothelial cells (H5V) were first exposed to 1.25 or 2.5µM UCB for 16h and then exposed to increasing doses of PA for 24h. As shown in Figure2 panel A and B, while PA treatment reduced cell viability by approximately 50% and increased cell death up to 20%, UCB pretreatment increased cell viability and reduced the cell death rate. Immortalized human proximal tubular epithelial cells (HK-2) were exposed to increasing Ang II concentration (0.01–10µM for 24h). Even if Ang II treatment affected podocytes cell viability, as expected (Baker and Kumar, 2006), there was no change on HK2 cell viability or cell mortality compared to control, when exposed to Ang II (Supplementary Figures S2A and S2B).

### PA Impact on CHOP Activation and IL-6 Release in Nj and jj Aortic Endothelial Primary Cells

**FIGURE 3:**
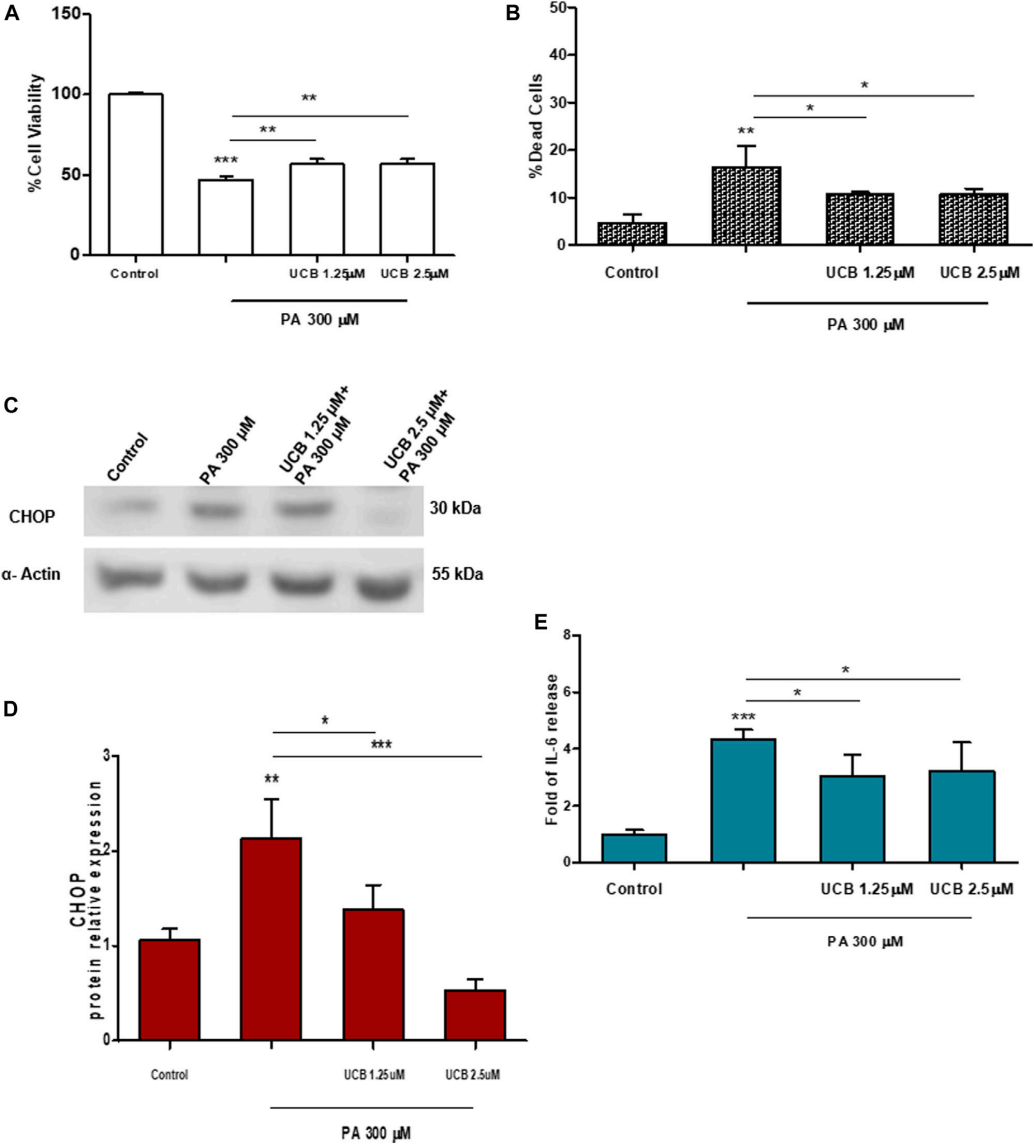
PA impact on CHOP activation and IL-6 release in normobilirubinemic (Nj) and hyperbiliruninemic (jj) aortic endothelial primary cells. Aortic endothelial cells were treated with PA 300 or 450μM or DMSO (as control) in the presence of BSA 75μM for 24h. **(A)** Representative Western Blot for CHOP and α-Actin. **(B)** The optical density of each band was normalized to α-Actin and represented as relative to control cells. **(C)** ELISA quantification of IL-6 release in the cell medium. Results were expressed as relative to control cells release, considered as one. Data were expressed as mean ± SD of at least three independent experiments. Significativity was calculated compared to the control or between samples indicated with bars at each sampling concentration point. *p < 0.05, **p < 0.01, ***p < 0.001.

We investigated the metabolic pathways involved in aortic endothelial cell viability focusing on ER stress and inflammation. Primary endothelial Nj and jj cells were treated with PA 300 and 450μM for 24h. A significant up-regulation of CHOP protein was observed after 24h of incubation with PA 300 and 450μM only in Nj aortic endothelial cells (*p* < 0.05). Conversely, the same treatment on jj showed a significant decrease in CHOP protein level (*p* < 0.05) (Figures 3A,B). A boost in IL-6 production upon PA 300µM and PA 450µM treatments was observed only in Nj cells, while cells from jj rats showed a significant decrease in IL-6 release (*p* < 0.001). The IL-6 release in the cell medium by jj cells was significantly lower than that released by cells from Nj rats (*p* < 0.001) (Figure 3C).

Similar results were observed in H5Vcells. Cells were first treated with UCB1.25 or 2.5µM for 16h and then exposed to PA 300µM for 24h. 300μMPA treatment significantly increased the expression level of CHOP protein (*p* < 0.01) while UCB pretreatment restores it to the control level (Figures 2C,D). Similarly, UCB pretreatment significantly (*p* < 0.05) reduced PA 300µM increase of IL-6 release (Figure 2E).

### Angiotensin II Impact on Apoptosis Activation in Nj and jj Podocyte Primary Cells

**FIGURE 4:**
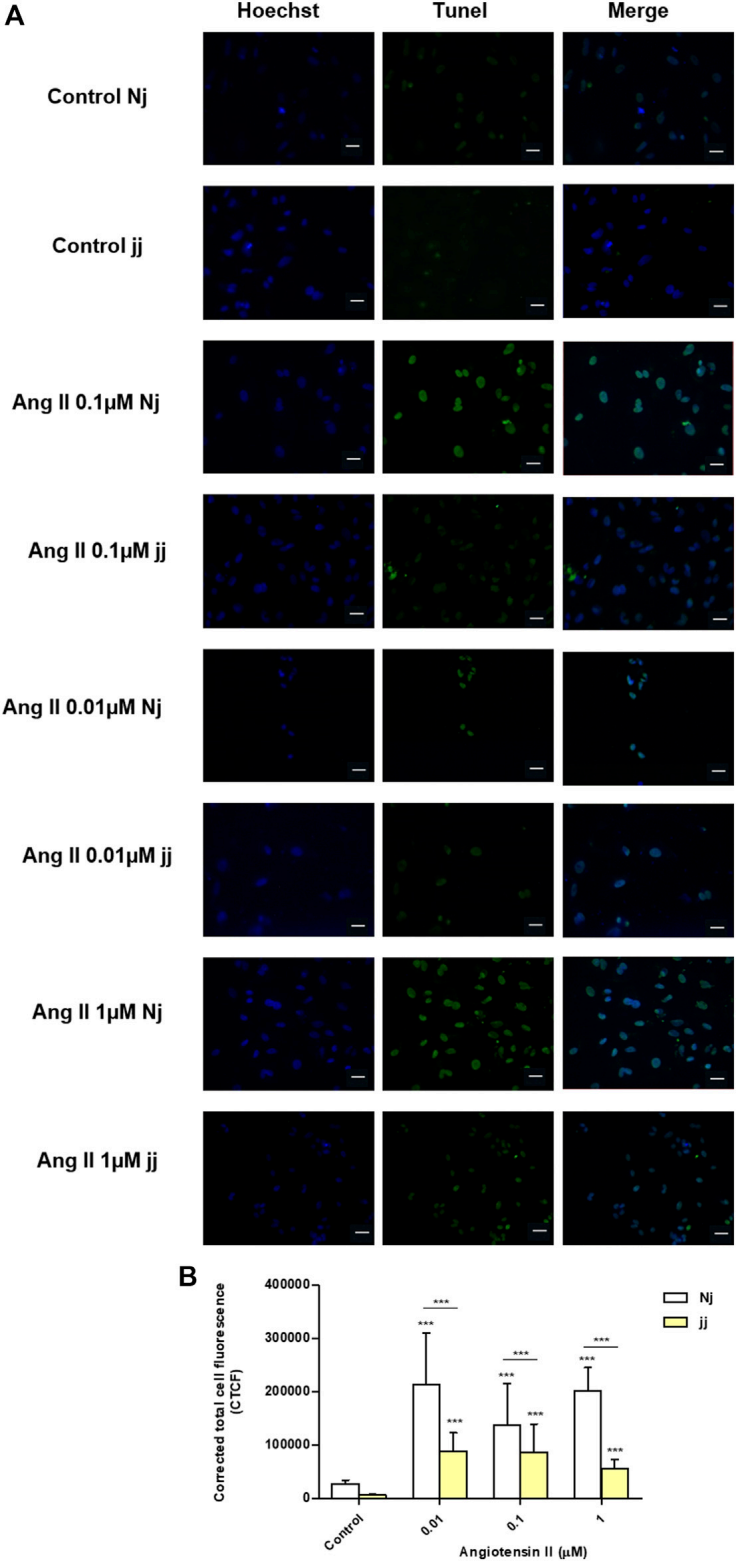
TUNEL staining of normobilirubinemic (Nj) and hyperbiliruninemic (jj) Gunn rat primary podocytes exposed to Ang II treatments. Primary podocytes were exposed to Ang II 0.01, 0.1, and 1µM for 24h. Cells treated with complete growth medium were considered as control. **(A)** Apoptotic cells were detected by TUNEL assay. Green fluorescence indicated TUNEL-positive and blue indicated Hoechst nuclear dye. Magnification ×40. Scale bar 50µm. **(B)** Quantification of fluorescence intensity. The fluorescence intensity was quantified using ImageJ and displayed as corrected total cell fluorescence (CTCF). Results shown represented the mean ± SD of three independent experiments. Significativity was calculated compared to the control or between samples indicated with bars at each sampling concentration point. *p < 0.05, **p < 0.01, ***p < 0.001.

**FIGURE 5:**
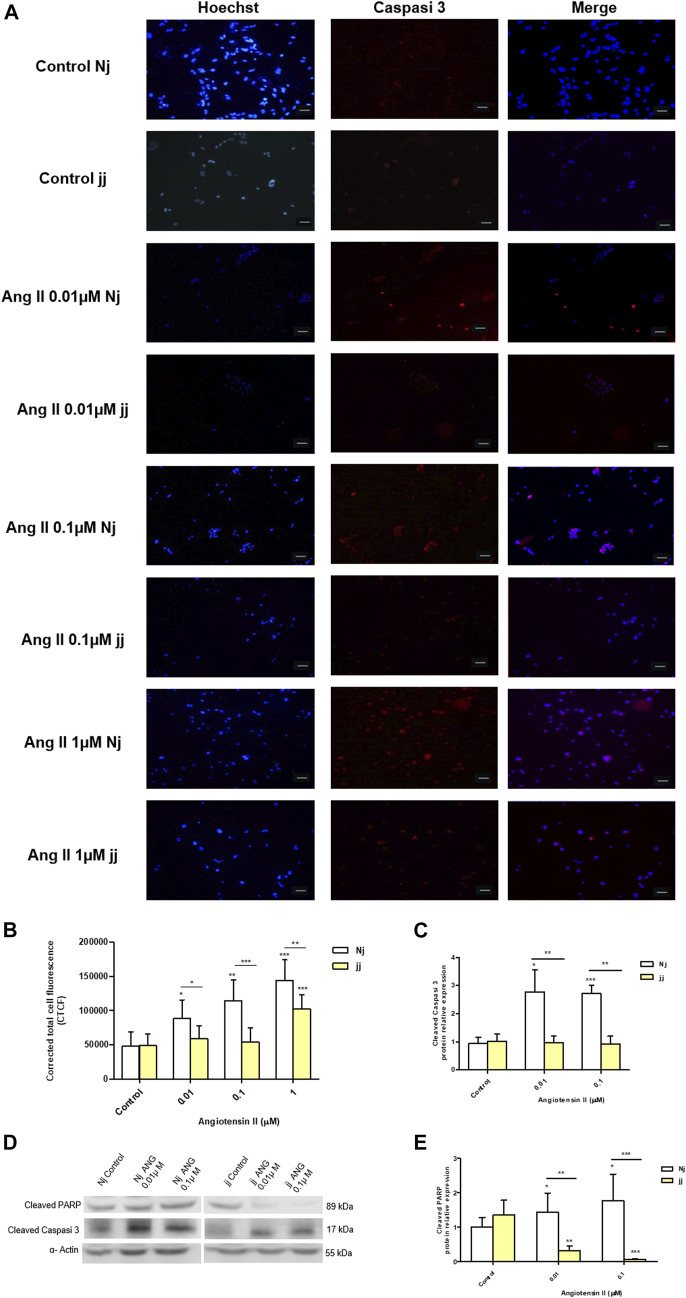
Activation of apoptosis signaling by Ang II in normobilirubinemic (Nj) and hyperbiliruninemic (jj) podocytes primary cells. Primary podocytes were exposed to Ang II 0.01, 0.1, and 1µM for 24h. Cells treated with complete growth medium were considered as control. **(A)** Representative immunofluorescence (*n* = 3) images showing immunostaining for cleaved Caspase-3 (red) and Hoechst (blue) for nuclei. Magnification ×20. Scale bar 50µm. **(B)** Quantification of Cleaved Caspase-3 fluorescence intensity. The fluorescence intensity was quantified using ImageJ and displayed in corrected total cell fluorescence (CTCF). **(C)** Representative Western blot analysis of cleaved PARP, cleaved caspase-3 and α-Actin expression in total cell lysates. **(D)** The optical density of cleaved caspase-3 protein from three independent experiments was normalized to α-Actin and represented as relative to untreated cells. **(E)** The optical density of cleaved PARP protein from three independent experiments was normalized to α-Actin and represented as relative to untreated cells. Significativity was calculated compared to the control or between samples indicated with bars at each sampling concentration point. *p < 0.05, **p < 0.01, ***p < 0.001.

Treatment with Ang II 0.01, 0.1, and 1µM for 24h showed a significant increase in the number of apoptotic podocytes in both genotypes, as detected by TUNEL assay (Figure 4A). However, the extent of Ang II-induced apoptosis was greater in podocytes harvested from Nj rats compared to the podocytes of jj rats at each Ang II concentration tested (Figure 4B). In normobilirubinemic podocytes, Ang II treatment induced a dose-dependent increase in cleaved caspase-3 expression assessed by immunofluorescence. Conversely, only 1µM Ang II significantly increased the expression of cleaved caspase-3 in jj podocytes (*p* < 0.001). Caspase-3 activation significantly (*p* < 0.01) differed between the two genotypes upon all Ang II treatments (Figures 5A,B). Due to the limited yield of total protein obtained from primary podocyte cultures, WB analysis was performed only on Ang II 0.01 and 0.1µM treatments for cleaved PARP and cleaved caspase-3 (Figure 5C). Both treatments induced an almost two-fold increase in the protein level of activated caspase-3 only in cells from Nj rats, while the expression remained similar to control levels in podocytes from jj rats (Figure 5D). The cleaved PARP expression was slightly induced in podocytes from Nj rats, while its expression is reduced compared to control cells in podocytes from jj rats (Figure 5E).

### Impact of UCB on Fibrosis Induced by Angiotensin II in Proximal Tubular Epithelial Cells

**FIGURE 6:**
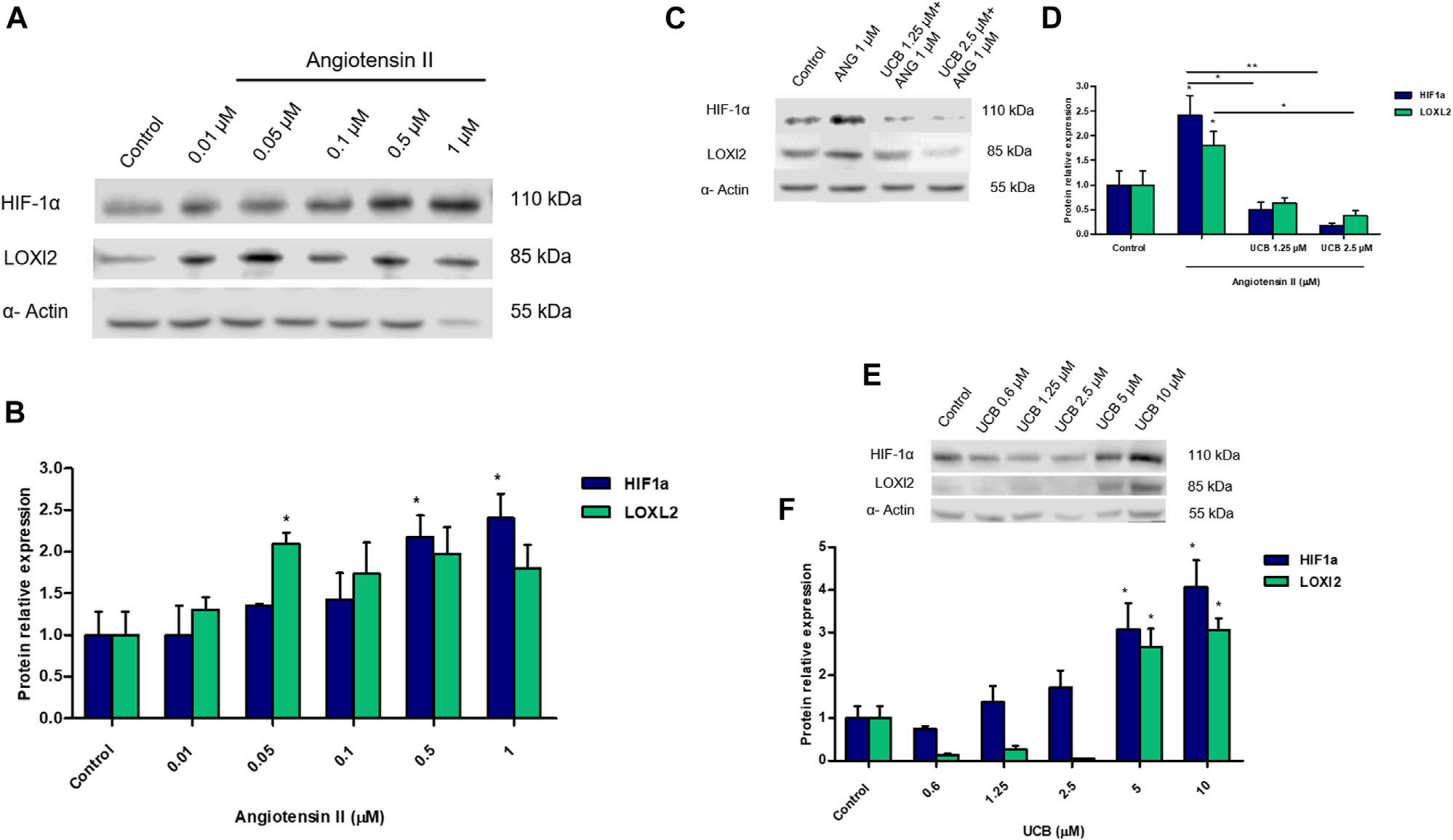
The impact of UCB on proximal tubular epithelial cells fibrosis induced by Ang II. **(A)** Representative Western blot analysis of HIF-1a, LOXl2 and α-Actin expression in HK2 cells treated with increasing doses of Ang II for 24h. **(B)** Theoptical density of each band was normalized to α-Actin and represented as relative to control cells. **(C)** Representative Western blot analysis of HIF-1α, LOXl2 and α-Actin expression in HK2. Cells were pre-incubated with UCB 1.25 or 2.5μM or DMSO 0.1% in the presence of BSA 30µM for 16h. At the end of incubation, UCB was removed and cells treated with Ang II 1μM for 24h. **(D)** The optical density of each band was normalized to α-Actin and represented as relative to control cells. **(E)** Representative Western blot analysis of HIF-1α, LOXl2 and α-Actin expression in HK2 cells treated with increasing doses of UCB for 24h. **(F)** The optical density of each band was normalized to α-Actin and represented as relative to control cells. Data were expressed as mean ± SD of three independent experiments. Significativity was calculated compared to the control or between samples indicated with bars at each sampling concentration point. **p < 0.05, **p < 0.01, ***p < 0.001.

Ang II is known to induce fibrosis in tubular epithelial cells. A dose-dependent increase of HIF-1α and LOXl2 protein expression was detected on immortalized human proximal tubular epithelial cells (HK-2) exposed to increasing concentration of Ang II for 24h. Treatment with Ang II concentrations 0.5 and 1µM showed a significant (*p* < 0.05) up-regulation of HIF-1α protein expression while LOXl2 protein expression was significantly (*p* < 0.05) up-regulated by AngII 0.05µM (Figures 6A,B). Moreover, the effects of Ang II on HIF-1α mRNA expression did not show any variation compared to controls (data not shown). Considering the induction o fHIF-1α and LOXl2 protein by Ang II, we investigated whether UCB pretreatment may influence its expression. Pretreatment with 1.25µM or 2.5 UCB significantly reduced HIF-1α protein induction by AngII in a dose-dependent manner (*p* < 0.05, *p* < 0.01). UCB also significantly (*p* < 0.05) reduced LOXl2 protein to 0.5-fold of control cells (Figures 6C,D). The effect of UCB alone on HIF-1α and LOXl2 protein expression was evaluated by exposing for 24h HK2 cells to a dose-dependent UCB treatment (from 0.6 to 10µM). HIF-1α and LOXl2 expression was reduced by UCB treatment up to 2.5µM, while higher concentration induced their expression (Figures 6E,F).

## Discussion

Several studies showed the protective effect of bilirubin against oxidative-stress based disease. In the present work, we assessed if life-long hyperbilirubinemia and bilirubin-priming might significantly contribute to the protection against metabolic insult *in vitro*. To this goal, we used primary cells obtained from hyperbilirubinemic jj and normobilirubinemic Nj Gunn rats exposed to damaging agents. Primary cells from jj Gunn rats were more viable and less prone to damage compared to cells from Nj Gunn rats, suggesting a modulating activity on cellular signaling of a life-long hyperbilirubinemia. This conclusion was further supported by data obtained in immortalized cell lines pretreated with UCB (bilirubin-priming) and then exposed to damage.

Studies comparing the response to damage between primed and not primed UCB cells are scant. Recently Valaskova et al. showed that the cell viability of primary hepatocytes from jj Gunn rats exposed to TNF-α 100ng/ml was significantly higher than that of primary hepatocytes from Nj Gunn rats (*p* < 0.05) (Valaskova et al., 2019). Our results expand this observation as we assayed the viability of other primary cell types (podocytes and primary aortic endothelial cells) exposed to increasing concentration of a damaging stimulus (PA and Ang II, respectively). In both cell models and at any toxic agent concentration, the viability of cells derived from jj Gunn rats was significantly higher than that of Nj cells. In line with this, UCB pretreatment in H5V cells exposed to PA reduced the percentage of dead cells and increased cell viability compared to DMSO pretreated cells. Bilirubin activates various nuclear and cytoplasmic receptors, resembling the endocrine activities of hormonal substances. This is true both for the “classic” hepatic nuclear receptors, and for some lesser-explored receptors or other signaling molecules (Vítek, 2020). We previously showed that 24h of bilirubin-priming modulates the glutathione levels in neuroblastoma cells through the induction of the System Xc-, and this renders the cell less prone to oxidative damage (Giraudi et al., 2011).

Bilirubin exerts potent anti-inflammatory and immunomodulatory activities (Jangi et al., 2013) and the interrelation between UCB, ER-Stress, inflammation, and associated cascade of events has been demonstrated (Barateiro et al., 2012). An increasing body of evidence suggests that mildly elevated bilirubin level could control inflammation, both *in vitro* and *in vivo*, through the mechanisms involved in NF-κB signaling pathway inhibition as well as the activation of inflammasomes (Li et al., 2020) suppressing the production of pro-inflammatory cytokines (Liu et al., 2008; Adin et al., 2017). We observed a reduced CHOP expression and IL-6 release in jj aortic endothelial primary cells exposed to PA compared to cells from Nj rats. Besides, in immortalized H5V cell line pretreatment with UCB significantly reduced CHOP expression and IL-6 release induced by PA treatment. Of notice, the TNF-α-stimulated nuclear translocation of NF-kβ was inhibited by UCB in H5V cells (Mazzone et al., 2009). Co-treatment with UCB resulted in reducing ER-stress and the subsequent inflammatory response also in *in vitro* model of gut inflammation (Gundamaraju et al., 2019). The anti-inflammatory and anti-oxidative effects may likely contribute to the protective role of bilirubin on vascular damage (Inoguchi et al., 2016).

Diabetic nephropathy (DN) is a complication of diabetes mellitus and is the leading cause of end-stage renal disease. The final effects of diabetic nephropathy involve endothelial injury, tubule-interstitial fibrosis, and podocytes detachment and apoptosis (Maezawa et al., 2015). Bilirubin provided a nephroprotective effect in different *in vivo* models as showed by the finding that jj Gunn rats showed significantly lower creatinine compared with Wistar rats at day 5 in a cisplatin nephrotoxicity model (Barabas et al., 2008). In streptozotocin-induced diabetic damage, jj Gunn rats exhibited significantly less urinary albumin excretion, did not develop renal mesangial expansion, and expressed lower levels of TGF-β and fibronectin than diabetic Nj Gunn rats (Fujii et al., 2010). We found that, upon Ang II treatment, primary podocytes from jj Gunn rats showed lower DNA fragmentation, cleaved caspase-3, and cleaved PARP induction than primary podocytes from Nj Gunn rats. Also, on the cyclosporine (CsA)-induced nephropathy rat model, a significantly lower number of apoptotic cells was observed in the bilirubin-treated rat kidneys compared to controls, confirming the bilirubin anti-apoptotic effect (Oh et al., 2013).

Recent studies have proposed for HIF-1α downstream function of a pro-fibrotic signaling cascade stimulated by angiotensin II, leading to epithelial-mesenchymal transition and excessive collagen apposition (Wang et al., 2011). We found that Ang II acts as a pro-fibrotic marker inducing HIF-1α and LOXl2 protein expression in a dose-dependent manner. Expanding the previous evidence (Kim et al., 2014), we showed that bilirubin modulated the expression of the pro-fibrotic marker with double-side behavior. UCB treatment up to 2.5µM decreased HIF-1α and LOXl2 expression compared to control, while higher UCB concentration induced their expression This is due to the extent of intracellular UCB concentration which determines the UCB anti-or pro-oxidant effect (Bianco et al., 2020). HIF-1α and LOXl2 protein induction by Ang II was significantly reduced by UCB pretreatment of 1.25 or 2.5µM corresponding to an antioxidant intracellular UCB concentration (Bianco et al., 2020). Bilirubin has been shown to have an anti-fibrotic effect on cyclosporine (CsA)-induced nephropathy *in vivo*, given that bilirubin pretreatment significantly improved afferent arteriolopathy, tubulointerstitial fibrosis, and tubular injury compared to the CsA-only treated rats (Oh et al., 2013).

Our data suggest that life–long hyperbilirubinemia exposure and bilirubin-priming significantly contribute to increase cell viability and enhance responses to damage in *in vitro* models of atherosclerosis and DN. Collectively these findings further support the beneficial effect of bilirubin on different organs.

## Data Availability

The original contributions presented in the study are included in the article/Supplementary Material, further inquiries can be directed to the corresponding author.
