# Supplementary figures and images for: Life-Long Hyperbilirubinemia Exposure and Bilirubin Priming Prevent In Vitro Metabolic Damage
Source: Front Pharmacol. 2021 Mar 12;12:646953. doi: 10.3389/fphar.2021.646953 (PMC7994257; doi:10.3389/fphar.2021.646953)

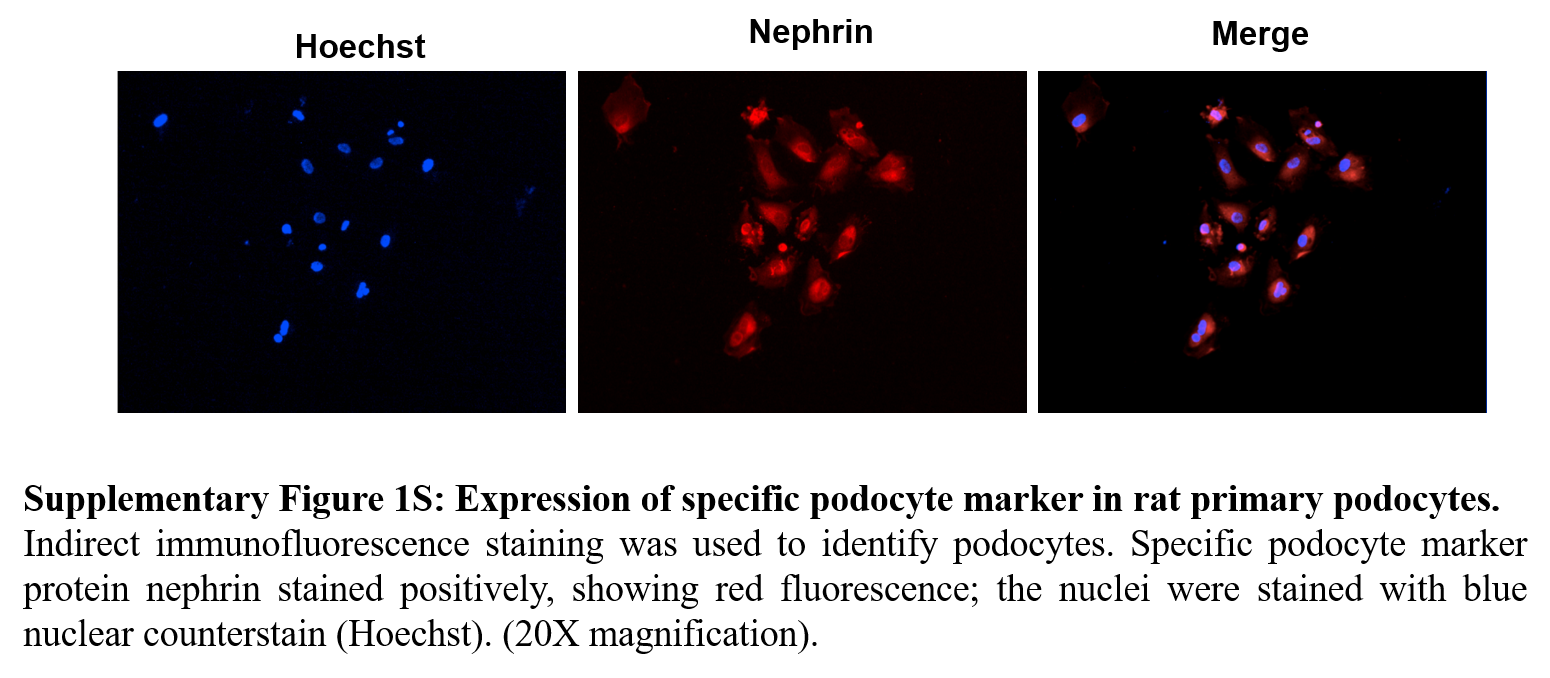

Supplement: Supplementary file 1 [file image1.tif]

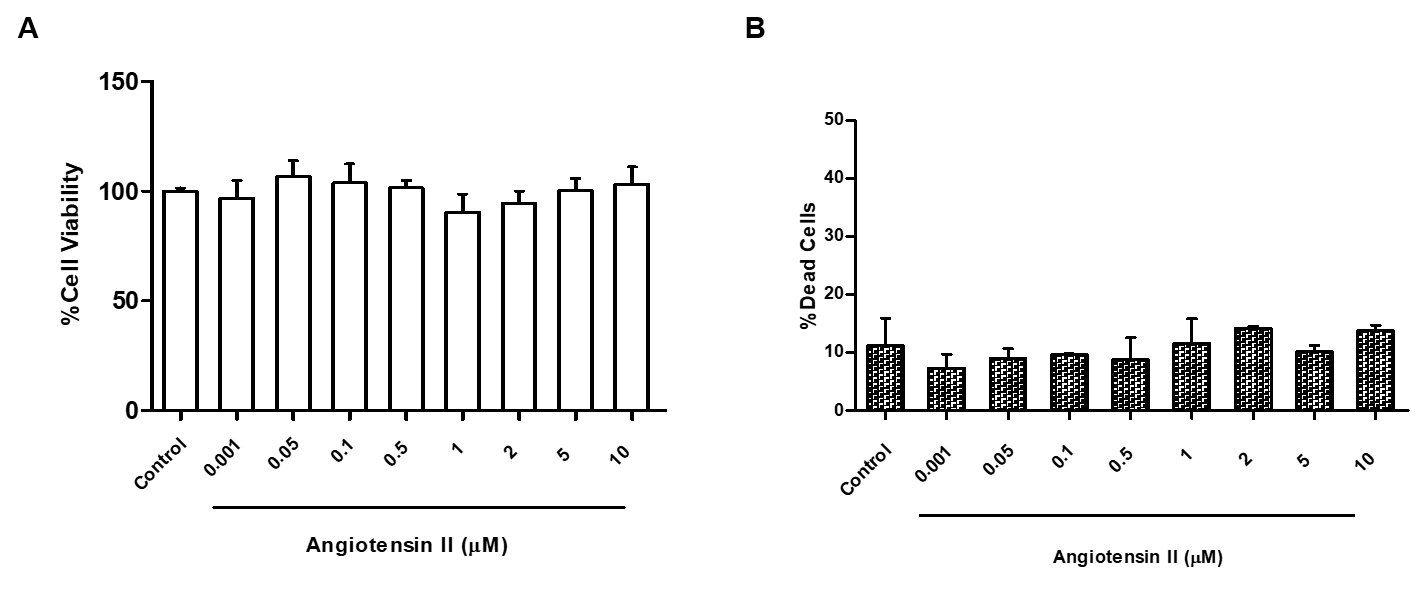

Supplement: Supplementary file 2 [file image2.tif]
